# Acute Effects of Growth Hormone on the Cellular Immunologic Landscape in Pediatric Patients

**DOI:** 10.7759/cureus.57383

**Published:** 2024-04-01

**Authors:** Jasmine Gujral, Brian A Kidd, Christine Becker, Eddye Golden, Hao-chih Lee, Seunghee Kim-Schulze, Mabel Yau, Joel Dudley, Robert Rapaport

**Affiliations:** 1 Pediatric Endocrinology, Yale School of Medicine, New Haven, USA; 2 Genetics, Icahn School of Medicine at Mount Sinai, New York, USA; 3 Genetics, Icahn School of Medicine at Mount Sinai, New York City, USA; 4 Immunology and Immunotherapy, Icahn School of Medicine at Mount Sinai, New York, USA; 5 Pediatric Endocrinology, Icahn School of Medicine at Mount Sinai, New York, USA

**Keywords:** mass cytometry, pediatric endocrinology, growth hormone stimulation test, clinical immunology, growth hormone deficiency

## Abstract

Introduction: Growth hormone (GH) and the immune system have multiple bidirectional interactions. Data about the acute effects of GH on the immune system are lacking. The objective of our study was to evaluate the acute effects of GH on the immune system using time-of-flight mass cytometry.

Methods: This was a prospective study of pediatric patients who were being evaluated for short stature and underwent a GH stimulation test at a tertiary care center. Blood samples for immunologic markers, i.e., complete blood count (CBC) and time of flight mass cytometry (CyTOF), were collected at baseline (T0) and over the course of three hours (T3) of the test. Differences in immune profiling in patients by timepoint (T0, T3) and GH response (growth hormone sufficient (GHS) versus growth hormone deficient (GHD)) were calculated using a two-way ANOVA test.

Results: A total of 54 patients (39 boys and 15 girls) aged five to 18 years were recruited. Twenty-two participants tested GHD (peak GH <10 ng/ml). The CyTOF analysis showed a significant increase from T0 to T3 in granulocyte percentage, monocyte count, and dendritic cell (DC) count; in contrast, a significant decrease was seen in T lymphocytes (helper and cytotoxic) and IgD+ B lymphocytes. The CBC analysis supported these findings: an increase in total white blood cell count, absolute neutrophil count, and neutrophil percentage; a decrease in absolute lymphocyte count, lymphocyte percentage, absolute eosinophil count, and absolute monocyte count. No significant differences were found between CBC/CyTOF measurements and GH status at either time.

Conclusions: This study provides the first high-resolution map of acute changes in the immune system with GH stimulation. This implies a key role for GH in immunomodulatory function.

## Introduction

There is growing evidence to substantiate a bidirectional cross-talk between the growth hormone (GH)-insulin-like growth factor-1 (IGF-1) axis and the immune system [1-3]. Growth hormone, GH-releasing hormone (GHRH), and IGF-1 are synthesized and secreted by various immune cells and organs [4-6]. In addition, GH, GHRH, and IGF-1 receptors are expressed in lymphoid cells and organs [7-10]. The GH receptor belongs to the cytokine superfamily of receptors, and the Janus kinase-signal transducer and activator of transcription pathway is common to both GH and cytokine signaling [11]. Hypophysectomy in rats caused severe GH deficiency, leading to severe immunodeficiency of the thymus-dependent cellular and antigen-induced humoral responses; these abnormalities were corrected after the administration of GH [12,13]. Studies show mild immunological dysregulation in humans with GH deficiency [14-17]. Growth hormone-deficient children treated with GH had a transient decrease in the percentage of B and T cells, soluble IL-2 receptor levels, and the response of lymphocytes to mitogenic stimulation; these changes were observed after a few weeks to months of therapy in vivo and in vitro [18-20]. However, the acute effects of GH on the human immune system are lacking. We conducted our study on pediatric patients undergoing GH stimulation testing and utilized their endogenous GH production to assess their acute cellular immune response. Immune responses were studied by performing CBC and time-of-flight mass cytometry (CyTOF) analyses on blood samples taken at the beginning and end of stimulation testing.

## Materials and methods

Study design and setting

This prospective study was approved by the Institutional Review Board of the Icahn School of Medicine at Mount Sinai, New York, NY, USA (approval no. IF2058362). The participants in the study were recruited at the Division of Pediatric Endocrinology and Diabetes at Mount Sinai Kravis Children’s Hospital in New York between September 2017 and March 2018. This study had three phases. Phase 1 included studying acute changes in the immune system induced by GH over three hours, which was done by utilizing the GH stimulation test. Phases 2 and 3 were designed to study chronic changes in the immune system after patients from phase 1 had been initiated on GH therapy. Phase 2 looked at data after six to nine months of GH therapy, and phase 3 looked at data after 12 to 15 months of GH therapy. This study describes Phase 1. 

The eligibility criteria for the study included pediatric patients aged five to 18 who were undergoing a GH stimulation test as part of an evaluation of short stature or growth failure. Short stature was defined as a height less than -1.5 standard deviations (SD) below the mean and less than -2 SD below the mid-parental height, and growth failure was defined as a height velocity less than -2 SD below the mean over one year [21]. The subjects were otherwise healthy. The exclusion criteria for the study were: patients unable to provide written consent from a parent or legal guardian; patients born small for gestational age (SGA) (birth length or birth weight below 2 SDs for gestational age); patients with known genetic syndromes; patients with chronic renal failure; and patients treated with corticosteroids or immunosuppressants within two months of the test. For all subjects, data collection included age, sex, height, height SDs, weight, weight SDs, race, ethnicity, birth weight, birth length, and growth velocity (GV). The BMI was calculated as weight (in kilograms) divided by height (in meters) squared. The BMI SDs, GV SDs, and IGF-1 SDs were also calculated. The pubertal status using Tanner staging was assessed and documented by a pediatric endocrinologist. 

The GH stimulation testing was done in the morning following a minimum overnight eight-hour fast. Participants received provocative agents: glucagon (1 mg) intramuscular injection and arginine hydrochloride (0.5 g/kg) intravenous infusion over 30 minutes [22]. Serum samples were obtained at baseline (T0) and 30, 60, 90, 120, 150, and 180 minutes (T3). The GH concentrations were measured at all time points via the double-antibody radioimmunoassay method by Esoterix Laboratory (Endocrine Sciences, Calabasas, CA, USA). An additional 5.5 ml of whole blood was taken for CBC and CyTOF analyses at T0 and T3. A stimulated peak GH level lower than 10 ng/ml for both provocative agents was considered GH deficient [23]. Subjects were then classified into a GH deficient group (GH <10 ng/ml) and a GH sufficient group (GH ≥10 ng/ml). All blood samples were collected by a clinician overseeing the GH stimulation test. Whole blood was stored in a liquid nitrogen tank until immune-based assay commencement.

CBC and CyTOF analysis

The CBC analysis was done using an automated hemocytometer (INCYTO C-chip, Incyto, Cheonan-si, Chungnam-do, KOR). A total of 17 CBC parameters were tested at both T0 and T3 and included total WBC count, absolute neutrophil count (ANC), neutrophil percentage, absolute lymphocyte count, lymphocyte percentage, absolute eosinophil count, eosinophil percentage, absolute monocyte count, monocyte percentage, absolute basophil count, basophil percentage, RBC count, hematocrit, mean corpuscular volume (MCV), red cell distribution width (RDW), platelet count, and mean platelet volume (MPV). For the CyTOF analysis, whole blood was stained using a panel of antibodies validated at the Human Immune Monitoring Center at Mount Sinai (New York, NY, USA). A total of 29 cell surface markers were used, as listed in Table 1.

**Table 1:** The 29 antibody panels used to stain PBMCs for CyTOF analysis. PBMCs: Peripheral blood mononuclear cells, CyTOF: Time of flight mass cytometry

Target	Metal label	Manufacturer	Clone	Catalog#
CD45	89Y	Fluidigm	HI30	3089003B
CD57	113In	Biolegend	HNK-1	359602
CD11c	115In	Biolegend	Bu15	337202
IgD	141Pr	Biolegend	IA6-02	348202
CD19	142Nd	Miltenyi	REA675	130-122-301
CD45RA	143Nd	Miltenyi	REA562	130-122-292
CD4	145Nd	Miltenyi	REA623	130-122-283
CD8	146Nd	Miltenyi	REA734	130-122-281
pSTAT5	147Sm	Fluidigm	47	3147012A
CD16	148Nd	Miltenyi	REA423	130-108-027
CD1c	150Nd	Miltenyi	REA694	130-122-298
CD123	151Eu	Miltenyi	REA918	130-122-297
CD66B	152Sm	Miltenyi	REA306	130-108-019
pSTAT1	153Eu	Fluidigm	4a	3153005A
CD86	154Sm	Biolegend	IT2.2	305449
CD27	155Gd	Miltenyi	REA499	130-122-295
Pp38	156Gd	Fluidigm	D3F9	3156002A
pSTAT3	158Gd	Fluidigm	4/P-STAT3	3158005A
pMAPKAP2	159Tb	Fluidigm	27B7	3159010A
CD14	160Gd	Miltenyi	REA599	130-122-290
CD56	161Dy	Miltenyi	REA196	130-108-016
CD169	166Er	Biolegend	7-239	346002
pERK1	167Er	Fluidigm	D13.14.4E	3167005A
CD3	168Er	Miltenyi	REA613	130-122-282
CD38	170Er	Miltenyi	REA671	130-122-288
CD161	171Yb	BioLegend	HP-3G10	339902
HLADR	174Yb	Miltenyi	REA805	130-122-299
pS6	175Lu	Fluidigm	N7-548	3175009A
CD11b	209Bi	Fluidigm	ICRF44	3209003B

**Figure 1:**
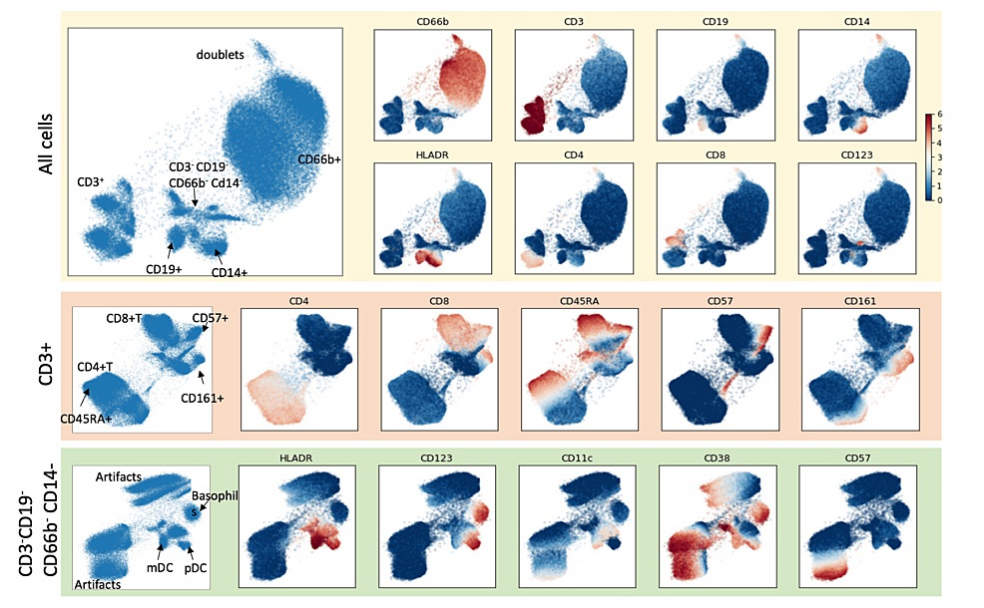
Characterization of immune cells from whole blood The t-distributed stochastic neighbor embedding (tSNE) plot of 1M cells acquired by CyTOF shows the phenotypic distribution of immune cells aggregated across samples. The tSNE plots are colored by marker intensity using cell-type specific markers: neutrophils and eosinophils (CD66b+), T-cells (CD3+), B-cells (CD19+), monocytes (CD14+) and others (CD66b-, CD3-, CD19- and CD14-). The CD3+ T cells and CD3-CD19-CD66b-CD14- cells were each separately subsetted for further visualization and analysis by tSNE. Within each subset, the tSNE plots are colored by cell-subset-specific marker intensities. tSNE: t-distributed stochastic neighbor embedding, CyTOF: Time of flight mass cytometry

To investigate populations of immune cells, we conducted t-distributed stochastic embedding (tSNE). Data clouds observed in the tSNE visualization indicate several known sub-populations: neutrophils and eosinophils (CD66b+), T cells (CD3+), B cells (CD19+), monocytes (CD14+), and others (CD66b-, CD3-, CD19- and CD14-) (Figure 1).

**Figure 2:**
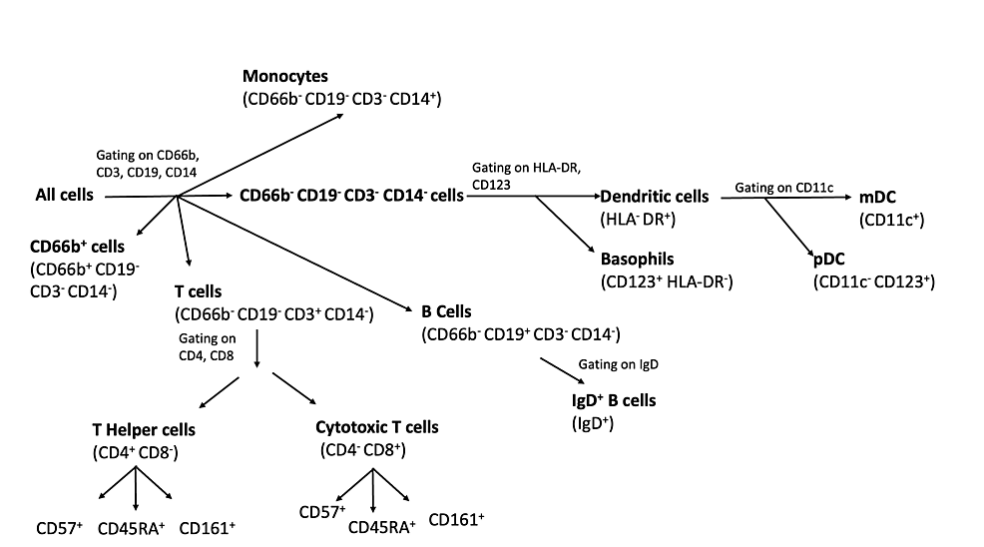
Gating strategy to define cell populations in CyTOF analyses CyTOF: Time of flight mass cytometry

We further performed a focused visualization of each sub-population. Within the T cell population, the tSNE plots depict the helper T cells (CD4+) and cytotoxic T cells (CD8+), which can be further divided into CD45RA+, CD57+, and CD161+ sub-populations. Within the CD66b-/CD3-/CD19-/CD14- population, we also observed distinct subpopulations that can be identified as myeloid dendritic cells (DCs) (HLA-DR+/CD11c+), plasmacytoid DCs (HLA-DR+/CD11c-/CD123+), and basophils (HLA-DR-/CD123+). Based on this unbiased observation, we designed a gating strategy (Figure 2) to determine the abundance of each subpopulation. We calculated the relative abundance of a cell subpopulation by using a ratio of cell subpopulation to total WBC count.

Statistical analysis

Demographic data, clinical features, and laboratory findings for each participant were manually entered by a clinician into the Research Electronic Data Capture (REDCap) system at Mount Sinai. Data were subsequently processed and cleaned, and quality control procedures were performed. A paired t-test (one-tailed, two-sample unequal variance) was conducted to compare clinical data between GH-deficient and GH-sufficient patients. For CBC and CyTOF, a two-way ANOVA test with repeated measures over time was done considering the interaction between GH status (GHD, GHS) and timepoint (T0, T3). The CyTOF analyses were done using a linear mixed model using R software (RStudio Inc., Boston, MA, USA). A p-value of 0.05 or less was chosen as the level of statistical significance.

## Results

Cohort characteristics

**Figure 3:**
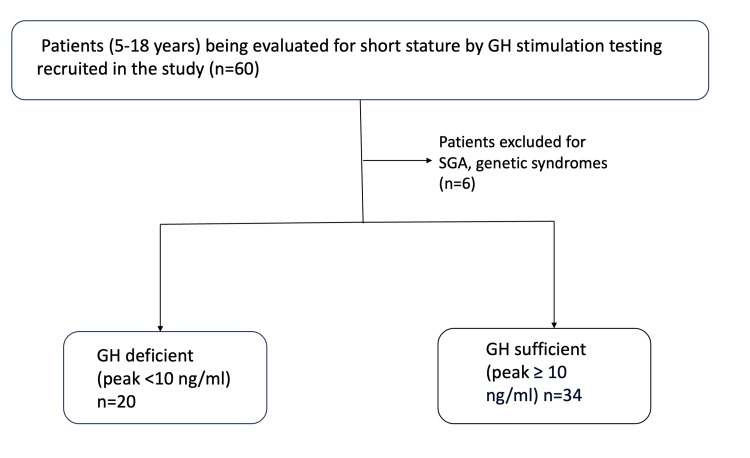
Study design GH: Growth hormone, SGA: Small for gestational age

In total, 60 patients were recruited for the study, and six were excluded, given their SGA status and genetic syndromes (Figure 3). Their clinical characteristics are summarized in Table 2. Of 54 patients, 62.9% (n = 34) were GHS, and 37% (n = 20) were GHD. Groups were not statistically different in age, race, height SDs, weight SDs, pubertal status, or IGF-1 SDs. However, they were statistically different in gender, BMI percentile, growth velocity, and peak GH values.

**Table 2:** Clinical characteristics of subjects in GHD and GHS groups *Parameters with significant p-values GH: Growth hormone, GHD: Growth hormone deficient, GHS: Growth hormone sufficient

Characteristics	GHS group	GHD group	p-value
Number (n)	34 (62.9%)	20 (37%)	
Mean age (years)	12.2 +/- 2.56	11.5 +/- 2.71	0.174
Gender	22 males (64.7%)	17 males (85%)	0.044*
	12 females (35.3%)	3 females (15%)	
Race	29 Caucasian (85.3%)	19 Caucasian (95%)	0.350
	4 Hispanic (11.7%)		
	1 African American (2.9%)	1 African American (5%)	
Height SDs	-1.69 +/ 0.64	-1.42 +/- 0.98	0.138
Weight SDs	-1.14 +/- 0.85	-0.66 +/- 1.29	0.075
BMI percentile	42.7 +/- 25.9	57.4 +/- 27.8	0.031*
Pubertal status	22 pubertal (64.7%)	10 pubertal (50%)	0.152
	12 pre-pubertal (35.2%)	10 pre-pubertal (50%)	
Growth velocity (cm/year)	5.07 +/- 2.26	3.77 +/- 1.04	0.004*
IGF-1 SDs	-0.67 +/- 1.1	-1.04 +/-1.07	0.117
Peak GH value (ng/ml)	20.7 +/- 11.66	6.99 +/- 2.3	<0.001*

CBC analysis

**Figure 4:**
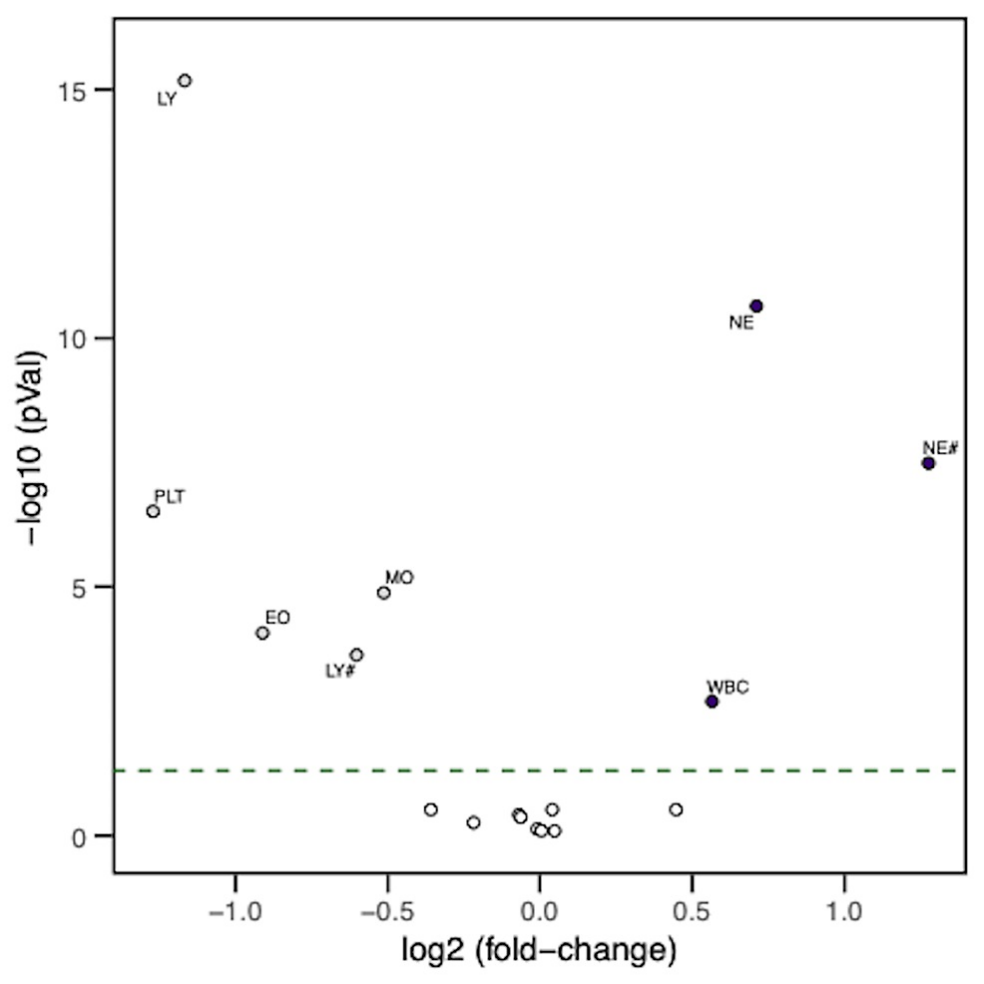
Volcano plot for CBC parameters The volcano plot shows changes in CBC parameters as a measure of large magnitude fold changes (x-axis) as well as high statistical significance (y-axis, -log10 of p-value). The dashed line represents the p-value cut-off (0.05), with the parameters above the line showing significant changes (p<0.05). The values to the right side of zero (WBC count, neutrophil percentage (NE#), and ANC count (NE)) show a significant increase from T0 to T3, and the values to the left of zero (lymphocyte percentage (LY#), lymphocyte count (LY), eosinophil count (EO), monocyte count (MO), and platelet count (PLT)) show a significant decrease from T0 to T3. T0: At baseline, T3: Over the course of three hours

We analyzed the differences in CBC parameters between T0 and T3 in the patients. Out of the 17 parameters analyzed, eight showed significant (p-value <0.05) changes between T0 and T3 (Figure 4, Table 3). Total WBC count, neutrophil percentage, and ANC significantly increased from T0 to T3, whereas lymphocyte percentage, lymphocyte count, eosinophil percentage, monocyte percentage, and platelet count significantly decreased from T0 to T3. There were no significant changes in eosinophil count, monocyte count, basophil percentage, basophil count, RBC count, hematocrit, MCV, RDW, or MPV. There were no statistically significant differences in any CBC parameters between the GHS and GHD groups at T0 or T3.

**Table 3:** Statistically significant changes in CBC parameters from T0 to T3. No differences were seen between the GHD and GHS groups. T0: At baseline, T3: Over the course of three hours, GHD: Growth hormone deficient, GHS: Growth hormone sufficient

Cell count/percentage	Change from T0 to T3	p-value
Total white blood cell count	Increased	0.002
Absolute neutrophil count	Increased	<0.001
Neutrophil percentage	Increased	<0.001
Absolute lymphocyte count	Decreased	<0.001
Lymphocyte percentage	Decreased	<0.001
Absolute eosinophil count	Decreased	<0.001
Absolute monocyte count	Decreased	<0.001
Absolute platelet count	Decreased	<0.001

CyTOF analysis

**Figure 5:**
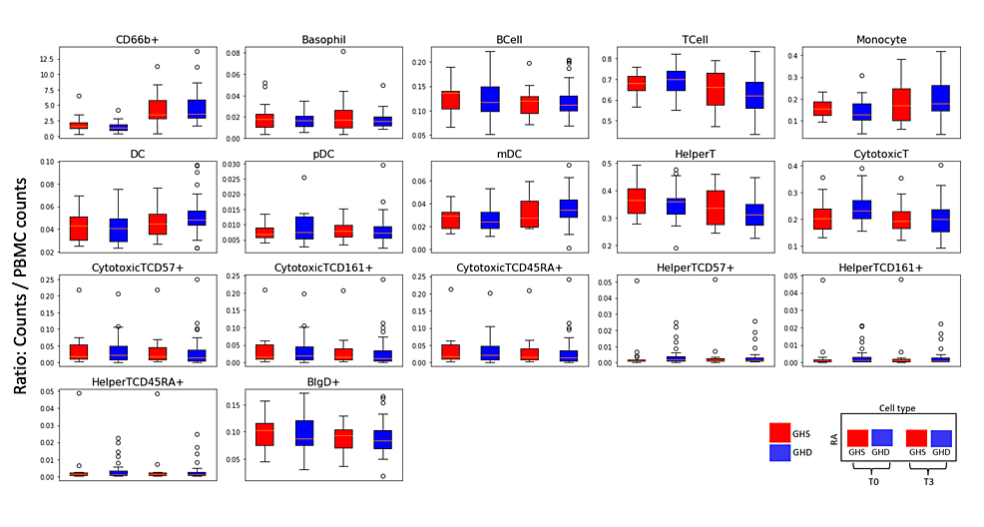
Changes in cell populations from T0 to T3 Relative abundance (RA) is defined as the ratio of the cell subpopulation count divided by total WBC counts. Red represents GHS patients, and blue represents GHD patients. The data pair on the left side of the graph represents the baseline (T0), and the data pair on the right represents the three-hour value at the end of the GH stimulation test (T3). The index template is displayed in the bottom right corner. Eight of the 15 cell subtypes show significant changes. Total T cells, helper T cells, cytotoxic T cells, and IgD+ B cells decreased from T0 to T3, while CD66b+ cells (neutrophils and eosinophils), monocytes, DCs, and myeloid DCs increased from T0 to T3. T0: At baseline, T3: Over the course of three hours, GHD: Growth hormone deficient, GHS: Growth hormone sufficient, DC: Dendritic cell

**Figure 6:**
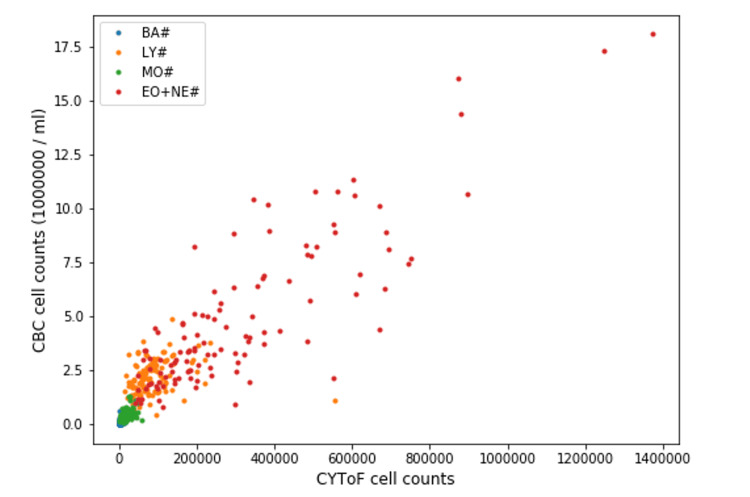
Linear relationship between CBC and CyTOF counts as measured in four cell populations BA: Basophils, LY: Lymphocytes, MO: Monocytes, EO +NE: Eosinophils and neutrophils, CyTOF: Time of flight mass cytometry

Cellular abundance (cell subtype population divided by total WBC count) showed a statistically significant (adjusted p-value <0.05) increase in CD66+ cells (sum of neutrophils and eosinophils), monocytes, and DCs from T0 to T3 and a significant (adjusted p-value <0.05) decline in both T and IgD+ B lymphocyte count (Figure 5, Table 4). The significant increase in DC frequency is largely due to myeloid DCs, while the decrease observed in T-cell frequency is contributed to by both cytotoxic T cells (CD8+) and helper T cells (CD4+). There were no statistically significant changes in basophil count, total B cell count, or plasmacytoid DCs. We found no significant differences in cellular abundance between the GHD and GHS groups. We observed a linear relation between cell counts measured by CBC and CyTOF in four general cell populations, namely lymphocytes, the sum of eosinophils and neutrophils, monocytes, and basophils (Figure 6).

**Table 4:** The CyTOF results demonstrate statistically significant changes from T0 to T3 in the relative abundance of various cell subtypes. No differences were seen between the GHD and GHS groups. CyTOF: Time of flight mass cytometry, T0: At baseline, T3: Over the course of three hours, GHD: Growth hormone deficient, GHS: Growth hormone sufficient

Cell subtype	Change from T0 to T3	p-value
CD66b^+ ^ (neutrophils + eosinophils)	Increased	<0.001
Total T cells	Decreased	<0.001
Monocytes	Increased	<0.001
Dendritic cells	Increased	<0.001
Myeloid dendritic cells	Increased	<0.001
Helper T cells	Decreased	<0.001
Cytotoxic T cells	Decreased	<0.001

## Discussion

This study demonstrates, for the first time, that GH stimulation results in acute changes in the cellular immune landscape in pediatric patients over three hours. We utilized endogenous GH production during a standard pediatric three-hour GH stimulation test in combination with CBC and CyTOF techniques to show significant changes in multiple immune cell compartments. We identified an increase in total WBC count, neutrophil count, monocyte count, and myeloid DC count, as well as a decrease in total lymphocyte count that includes both T cells (CD4+ and CD8+) and IgD+ B cells. These findings are similar to those described by Rapaport et al. in a cohort of children with GH deficiency who were found to have decreased total T lymphocytes and percent B lymphocytes after 12 months of GH treatment [20]. Our study shows that GH plays a role in immune regulation and acutely alters immune cell composition.

In both in vitro and in vivo studies, GH has been shown to cause proliferation and differentiation of erythroid and myeloid progenitor cells, increase monocyte migration, increase neutrophil adhesion, and increase cytokine production; it also enhances lymphocyte development, immunoglobulin production, and activity of natural killer cells and macrophages, along with respiratory and oxidative burst activity of granulocytes [24-30]. In a study conducted by Pagani et al., in 13 prepubertal GHD children, there was a significant increase in proinflammatory cytokines IL-6 and TNFα levels in circulation six hours after their first GH injection (0.23 mg/kg/week), supporting immune activation [31]. In a genome-wide DNA methylation study in men with congenital hypopituitarism, the genes that showed aberrant methylation included those implicated in the immune system, such as the regulatory-associated protein of mammalian target of rapamycin (mTOR) complex 1 [32]. In a study done on mice using recombinant IGF-1 for a period of seven and 14 days, it was shown that there was a decrease in peripheral lymphocyte counts and an increase in the neutrophil count from 33% to 48%, with an increase in the weights of both the thymus and spleen [33]. Since GH acts via an increase in serum IGF-1 levels, it could be postulated that some results of the shifts in immune cell compartments are indirectly mediated via IGF-1.

We identified a significant rise in the absolute neutrophil count at T3. In acromegalic patients and in vitro studies, it has been demonstrated that GH prevents neutrophil chemotaxis and increases polymorphonuclear neutrophil adhesion [30,34]. In a study of six adults with GHD who were given GH for a year, significant increases were seen in granulocyte-colony stimulating factor and neutrophil counts two months after GH initiation [35]. In another study, it was seen that SGA children developed persistent neutrophilia with GH administration [36]. In multiple studies, it has been shown that GH increases both T-cell adhesion to matrix proteins and T-cell migration [37]. This could imply a redistribution of T cells from the periphery to central lymphoid organs, causing a decrease in their numbers, as seen in our study. Our study showed an acute decrease in peripheral T cell and IgD+ B cell counts. However, peripheral lymphocyte counts are only a fraction of the total lymphocyte pool and do not reflect lymphocyte counts in central immune organs. Growth hormone therapy has been shown to have a major role in thymopoiesis when used over longer periods, as suggested by its use in immune reconstitution in patients with AIDS that show a marked increase in thymic mass and numbers of naïve CD4+ T cells after six months of GH therapy [38]. In our study, DC counts, particularly myeloid DCs, significantly increased with GH stimulation, whereas plasmacytoid DCs did not change significantly. This is concordant with other studies that show GH promotes the maturation and activation of DCs [39-41].

Other changes seen in our study include a significant decline in platelet counts from T0 to T3. In a study of seven patients with Laron syndrome (GH resistance), administration of IGF-1 for five years led to a decrease in platelet count. This could imply that changes in platelet redistribution begin acutely after GH [42]. Although GH is very well established as a hematopoietic agent, we did not see acute changes in the RBC counts in our study [43]. This is expected as GH acts on erythroid progenitor precursor cells rather than on peripheral blood, and the process of erythropoiesis takes weeks [26].

The two groups (GHD vs. GHS) were not found to be statistically different concerning CBC or CyTOF changes. Perhaps a lower level of GH (<10 ng/ml) and/or the cumulative effect of GH stimulation, even at smaller amplitudes, over three hours is sufficient to cause changes detected in the GHD group. Also, the deficiency cut-off of 10 ng/mL is not reflective of severe GHD. Growth hormone stimulation testing is known to have poor specificity and reproducibility, and a substantial percentage of healthy children, as well as children with obesity and pre-pubertal status, may falsely test below the accepted cut-off [44].

A limitation of our study is that we are utilizing peripheral cell lines that may not fully represent the central immune organs; for example, peripheral lymphocytes only represent a small fraction of the total lymphocyte pool. A confounding factor that needs to be considered is whether the provocative agents, i.e., arginine and glucagon, or the stress of the procedure itself (intravenous line placement) could have contributed to these changes. Having age- and sex-matched controls would be ideal to remove this potential confounder. Our sample size of 54 patients is relatively small; a larger sample size can increase the power of the study. We have also not studied the functionality of the immune cells, such as responsivity to mitogens or immunoglobulin levels, which could provide further insight into the changes in the immune cellular functions. The study's strengths are a relatively homogenous population regarding race (mostly Caucasian), data from two time points; hence, each patient serves as his or her control, and a mass cytometry technique that allows deep immune cell phenotyping with improved accuracy and minimal background signal. 

Despite laboratory evidence of changes induced by GH, children with GH deficiency do not show obvious clinical signs of immune dysfunction. This may be due to local GH production by the immune cells inducing autocrine and paracrine effects and compensating for the lack of pituitary GH. Human studies have not been done to study immune dysfunction with a lack of local production of GH, its receptors, and/or IGF peptides. The absence of obvious immune dysfunction could also be explained by the presence of detectable, albeit lower, levels of GH as opposed to negligible GH, as seen in hypophysectomized rats, where the immune dysfunction is more marked [45]. Growth hormone may not be an obligate factor in the immune cell development-regulation pathway but rather has more of a supplemental or synergistic role.

## Conclusions

In summary, we have demonstrated with the help of CyTOF and CBC analyses that GH causes significant measurable perturbations in the cellular immune system in vivo over a short period of three hours, implying that it has an immunomodulatory function. Whether these changes persist chronically or not is unknown and will be examined in the second and third phases of our study. The potential use of GH as a diagnostic and therapeutic tool could be explored in patients with immune deficiencies. Finally, given the varied and not yet fully understood immunologic effects of GH, clinicians need to caution against the indiscriminate use of GH. Further prospective studies are required to confirm these findings.
